# Hemolytic jaundice induced by pharmacological dose ascorbic acid in glucose-6-phosphate dehydrogenase deficiency: A case report: Retraction

**DOI:** 10.1097/MD.0000000000018261

**Published:** 2019-11-27

The journal is retracting the article, “Hemolytic jaundice induced by pharmacological dose ascorbic acid in glucose-6-phosphate dehydrogenase deficiency: A case report”,^[1]^ which appears in Volume 97, Issue 51 of *Medicine*, after a reader concern about the article. The article does not support the hypothesis.
